# Fixation method, not vascular injury, is the primary determinant of union in gunshot-related femoral fractures: a retrospective cohort analysis

**DOI:** 10.1186/s13018-025-06605-2

**Published:** 2026-01-06

**Authors:** Enver İpek, Yusuf Altuntaş, Bahadır Balkanlı, İsmail Demirkale

**Affiliations:** Şişli Hamidiye Etfal Hospital Sarıyer, İstanbul, Turkey

**Keywords:** Femoral fractures, Gunshot wounds, Intramedullary nailing, External fixation

## Abstract

**Background:**

Gunshot-related femoral fractures pose significant challenges due to periosteal disruption, contamination, and potential vascular injury. The impact of fixation method and vascular status on union time and functional outcomes remains incompletely defined.

**Objectives:**

To evaluate the influence of vascular injury and fixation technique on fracture union and clinical outcomes in gunshot-induced femoral fractures. We hypothesized that intramedullary nailing (IMN) would demonstrate faster union than external fixation (EF), and that vascular injury would prolonged healing.

**Methods:**

A retrospective cohort of 74 patients treated between 2015 and 2024 was analyzed. Group 1 consisted of EF with vascular injury (*n* = 19), Group 2 of EF without vascular injury (*n* = 19), and Group 3 of IMN without vascular injury (*n* = 36). Clinical variables, fracture characteristics, operative details, and radiographic outcomes were recorded. Delayed union was defined as union time > 6 months. Statistical analyses included ANOVA, Tukey tests, Fisher’s exact tests, and multivariate regression.

**Results:**

The mean age was 32.1 ± 9.7 years, and 98.6% were male. Most fractures occurred in zone 2 (85.1%). The mean operative time was longest in Group 1 (319 ± 61.3 min) compared with Groups 2 and 3. Overall mean union time was 7.4 ± 3.7 months; Group 3 demonstrated significantly shorter time for union (5.6 ± 3.2 months) than Group 1 (9.5 ± 2.9 months) and 2 (8.7 ± 3.6 months) (*p* < 0.001). Knee ROM was greatest in Group 3 (125.1° ± 7.5°, *p* = 0.001). Delayed union occurred in 34/38 EF patients (89.5%) and 11/36 IMN patients (30.6%). EF was associated with a substantially higher risk of delayed union than IMN (OR 19.3, 95% CI 5.5–67.8; *p* < 0.001). Vascular injury also increased delayed union risk, occurring in 18/19 patients with vascular trauma versus 27/55 without (OR 18.7, 95% CI 2.3–149.7; *p* = 0.0003). Fixation method was the only independent predictor of union time in multivariate analysis (*p* < 0.001).

**Conclusion:**

IMN results in faster union, better alignment, and superior knee motion compared with EF in gunshot-related femoral fractures. Although vascular injury contributed to prolonged healing, fixation method had the dominant influence on outcomes. Prospective studies with larger cohorts are needed.

## Introduction

Gunshot injuries have become increasingly prevalent worldwide, primarily due to mass conflicts, interpersonal violence, criminal activity, and terrorism. The majority of these injuries (91%) occur in males, with an average age of 32 years [1]. Among firearm-related orthopedic traumas, femoral fractures are the most common, accounting for approximately 40% of cases [2, 3]. Although many civilian gunshot injuries to the femur are considered low-energy, they can still cause substantial damage to bone, muscle, and neurovascular structures, potentially leading to severe morbidity or even amputation.

The selection of an appropriate fixation method after a gunshot-related fracture depends on several factors, including skin integrity, contamination level, and the extent of bone loss. Based on these parameters, intramedullary nailing or external fixation may be preferred. Intramedullary nailing is widely accepted as an effective treatment for femoral fractures resulting from low-energy gunshot injuries [4], whereas rapid external fixation followed by vascular repair is generally recommended in cases involving concomitant vascular injury [5]. However, the direct effect of vascular repair on fracture healing remains insufficiently clarified, and only a limited number of studies have investigated how external fixation may delay fracture union compared with intramedullary nailing [6].

Therefore, this study was designed to evaluate the effects of vascular injury and the chosen fixation method on the healing process of femoral fractures caused by gunshot injuries. We aimed to determine whether the presence of vascular injury negatively influences fracture healing and whether intramedullary nailing provides advantages over external fixation regarding union time and complication rates. We hypothesized that both vascular injury and fixation type significantly affect fracture union outcomes.

## Materials and methods

This study was designed as a single-center retrospective cohort analysis. Institutional ethics committee approval was obtained prior to data collection (Approval No: 4752, dated 11/02/2024). All procedures were conducted in accordance with the Declaration of Helsinki. Medical records of patients treated for gunshot-related femoral fractures at Şişli Hamidiye Etfal Hospital between 2015 and 2024 were reviewed electronically using the prospectively collected hospitals’ records. Surgical parameters and follow-up documentation were also examined. A total of 117 eligible patients were identified.

Patients were categorized into three groups based on fixation method and presence of vascular injury. Group allocation was defined by maintaining a constant fixation type and differentiating according to vascular status: Group 1: Patients with vascular injury treated with external fixation, Group 2: Patients without vascular injury treated with external fixation and Group 3: Patients without vascular injury treated with intramedullary nailing.The inclusion criteria were age ≥ 18 years, femoral fracture resulting from gunshot injury, fracture located in the femoral diaphysis or metaphysis, minimum 12-month clinical and radiological follow-up and follow-up continued until clinical and radiographic union was achieved.Exclusion criteria were intra-articular femoral fractures, nonoperative treatment, use of alternative implants or revision procedures (including 22 plate-screw cases), incomplete clinical or radiological data, implant change during follow-up (21 cases; 13 conversions from external fixation to intramedullary nailing, 7 plate, 1 screw) and revision due to deep infection or pin-site infection (5 cases).

The fixation method was determined in the emergency department according to standard clinical algorithms. In all cases of vascular injury, a Limb Reconstruction System external fixator (LRS-EF) was applied first, followed immediately by vascular repair, ensuring consistency across Group 1. Patients without vascular injury underwent stabilization using either intramedullary nailing or LRS-EF depending on open fracture characteristics. All patients received standard perioperative antibiotic prophylaxis.

Demographic characteristics (age, sex, comorbidities, smoking status) and injury-related variables were recorded. Fractures were classified using the AO/OTA system and localized according to gunshot fracture zones [7]. Open fractures were graded according to the Gustilo-Anderson classification. Preoperative American Society of Anesthesiologists (ASA) scores, time from injury to surgery, operative duration, and associated neurovascular injuries were documented.

Following initial evaluation, all patients underwent irrigation, debridement, and antibiotic administration [8]. Patients with diminished peripheral pulses underwent angiographic assessment and surgical planning accordingly. Complications during follow-up (infection, implant failure, loss of reduction) were recorded.Acute limb shortening was not required in any patient. Limb length was preserved, and vascular continuity was restored using primary repair, end-to-end anastomosis, interposition grafts, or bypass grafting as indicated.

Fractures were anatomically classified using the AO/OTA system and categorized according to injury zones (Zone 1: proximal, Zone 2: diaphyseal, Zone 3: distal) [7]. Two orthopedic surgeons blinded to group allocation independently assessed all radiographs. Imaging obtained at injury, postoperatively, and during follow-up was evaluated for fracture characteristics and healing progression.Clinical union was defined as painless full weight-bearing.Radiographic union was defined as bridging callus visible in at least three cortices on orthogonal views. Inter-observer reliability was calculated using the intraclass correlation coefficient (ICC), with values > 0.80 indicating excellent agreement.Postoperative alignment was evaluated using final AP and lateral radiographs. Rotational alignment and limb shortening were assessed by comparing the injured and contralateral sides using the hip center–knee center axis. Sagittal and coronal angulations were measured and compared across groups [8].

### Statistical analysis

Statistical analyses were performed using SPSS version 25.0. Continuous variables were presented as mean ± SD. Distribution normality was assessed using the Shapiro–Wilk test, and variance homogeneity using Levene’s test.Group comparisons were made using one-way ANOVA with Tukey HSD post-hoc testing. A multivariate linear regression model was constructed to identify independent predictors of fracture union time, including age, BMI, ASA score, limb length discrepancy, angular deformity, and treatment group. Sex was excluded due to homogeneous distribution (98.6% Male). Treatment group remained a significant independent predictor (*p* < 0.05).To adjust for multiple comparisons, Bonferroni correction was applied (adjusted α = 0.0167).

## Results

Between 2015 and 2024, a total of 117 patients with gunshot-related femoral fractures were treated at our institution. After applying the predefined inclusion and exclusion criteria, 74 patients were included in the final analysis: 19 patients in Group 1 (external fixation with vascular injury), 19 patients in Group 2 (external fixation without vascular injury), and 36 patients in Group 3 (intramedullary nailing without vascular injury). The differences in group sizes resulted solely from eligibility based on clinical and radiological criteria, without any attempt to achieve numerical balance.

The mean age of the cohort was 32.1 ± 9.7 years, and 98.6% of the patients were male (*n* = 73). Fractures were more commonly located on the left side (48.6%), although right-sided fractures predominated in Groups 1 and 2, whereas left-sided fractures were more frequent in Group 3. Of them, 14 patients (18.9%) were smokers. Vascular injury was present in 19 patients (25.7%), and neurological injury in 14 patients (18.9%). The mean BMI was 26.5 ± 2.0 kg/m². The mean overall operative time was 215.6 ± 89.2 min, with Group 1 showing the longest procedures (319 min), followed by Group 3 (194 min) and Group 2 (153 min). Demographic and baseline clinical variables including age, BMI, side of injury, and smoking status did not differ significantly between the groups (*p* > 0.05). Sex distribution was not compared statistically because only one female patient was included (Table 1).

**Table 1:** Comparison of demographic, clinical, radiological, and surgical parameters among femoral fracture patients

	Group 1 (*n* = 19)	Group 2 (*n* = 19)	Group 3 (*n* = 36)	Total (*n* = 74)	*P* value
	Vascular injury, EF	No vascular injury, EF	No vascular injury, IMN		
Demographic and clinical data					
Age (years) (Mean ± SD)	31.7 ± 5.2	32.3 ± 10	32.2 ± 11.4	32.1 ± 9.7	0.81
Gender—Male (%)	100	100	97.2	98.6	
Fracture side -right (%)	12 (54.83%)	10 (52.63%)	14 (38.9%)	36 (48.6%)	0.213
BMI (kg/m² ) (Mean ± SD)	26.4 ± 1.9	26.3 ± 1.8	26.7 ± 2.1	26.5 ± 2.0	0.751
Smoking n, (%)	4 (21%)	3 (15.8%)	7 (19.4%)	14 (18.9%)	0.912
Vascular injury n (%)	19 (%100)	0	0	19 (%25.68)	
Fracture location (Ao classification)					
Proximal femur (AO 31) n (%)	0 (0.0%)	6 (31.6%)	4 (11.1%)	10 (%13.5)	
Femoral shaft (AO 32) n (%)	11 (57.9%)	7 (36.8%)	29 (80.6%)	47 (%63.5)	
Distal femur (AO 33) n (%)	8 (42.1%)	6 (31.6%)	3 (8.3%)	17 (%23)	
Injury zone					
Zone 1 n (%)	0 (0.0%)	2 (10.5%)	4 (11.1%)	6 (8.1%)	
Zone 2 n (%)	16 (84.2%)	15 (78.9%)	32 (88.9%)	63 (85.1%)	
Zone 3 n (%)	3 (15.8%)	2 (10.5%)	0 (0.0%)	5 (6.8%)	
ASA Score					
ASA 1 n (%)	16 (84.2%)	16 (84.2%)	32 (88.9%)	64 (86.5%)	
ASA 2 n (%)	2 (10.5%)	3 (15.8%)	3 (8.3%)	8 (10.8%)	
ASA 3 n (%)	1 (5.3%)	0 (0.0%)	1 (2.8%)	2 (2.7%)	
Radiological evaluations					
Varus-Valgus deformity (Mean ± SD)	4.62 ± 4	4.6 ± 2.9	3.26 ± 2.3	3.95 ± 3	0.155
Antecurvatum-Recurvatum deformity (Mean ± SD)	6.65 ± 5.4	6.49 ± 4.9	3.11 ± 2.2	4.89 ± 4.3	**0.002**
Limb length discrepancy	5.89	7.94	3.58	5.29	0.135
Knee joint range of motion (Mean ± SD)	116.5 ± 9.4	118.2 ± 8.8	125.1 ± 7.5	121.1 ± 9.1	0.037
Operative and follow-up data					
Time to surgery (hours)	1.37	60.63	54.67	42.52	
Operation time (minutes)	319	153	194	215.57	
Union time (weeks) (Mean ± SD)	40.5 ± 12.5	37.3 ± 15.6	24.1 ± 13.5	31.7 ± 15.6	**< 0.001**
Union time (months) (Mean ± SD)	9.5 ± 2.9	8.7 ± 3.6	5.6 ± 3.2	7.4 ± 3.7	**< 0.001**
Follow-up duration (years) (Mean ± SD)	6.5 ± 2.2	5.5 ± 3.1	4.5 ± 2.2	5.27 ± 2.6	0.019
Fasciotomy	11 (%57.89)	0	0	11 (%14.9)	
Neurological injury n (%)	7 (%36.8)	3 (%15.8)	4 (%11.1)	14 (%18.9)	

Values are given as Mean ± SD or n (%). *p* values < 0.05 were considered statistically significant. EF, external fixation; IMN, intramedullary nailing; ASA, American Society of Anesthesiologists classification; BMI, body mass index; n (%), number (percentage). Zone classification is based on anatomical fracture location in the femur (Zone 1: proximal, Zone 2: diaphyseal, Zone 3: distal)

Regarding fracture characteristics, 63.5% of fractures were located in the femoral shaft (AO 32), 23% in the distal femur (AO 33), and 13.5% in the proximal femur (AO 31). Overall, 85.1% of fractures occurred in Zone 2, with the majority being diaphyseal fractures. In Group 1, fractures were predominantly in Zones 2 (84.2%) and 3 (15.8%), with no proximal (Zone 1) injuries. Group 2 fractures were mainly located in Zone 2 (78.9%), and Group 3 fractures were primarily shaft fractures in Zone 2 (88.9%). According to the ASA classification, 86.5% of patients were classified as ASA 1 (Groups 1 and 2: 84.2%; Group 3: 88.9%). ASA 3 status was observed in one patient each in Groups 1 (5.3%) and 3 (2.8%), with no ASA 3 patients in Group 2.

Fasciotomy was required in 11 patients (57.9%) with vascular injury, all in Group 1, and wound closure was achieved after a period of follow-up. Group 1 patients underwent earlier surgical intervention and had significantly longer operative durations compared with the other groups. In Group 3, antegrade intramedullary nailing was used in 29 patients (80.6%), whereas retrograde nailing was performed in 7 patients (19.4%).

The mean follow-up duration for the entire cohort was 5.27 ± 2.6 years and was longest in Group 1. The mean knee joint range of motion was 121.1 ± 9.1°, with significantly greater motion observed in Group 3 compared with the other groups (*p* = 0.037; Table 1).

The mean coronal varus–valgus deformity was 3.95°, with no significant difference among the three groups (*p* = 0.155). The mean varus/valgus angles were 4.62° in Group 1, 4.60° in Group 2, and 3.26° in Group 3. The mean sagittal antecurvatum–recurvatum deformity angle was 4.89°, and alignment in the sagittal plane was significantly better in Group 3. The mean deformity angle was 3.1° in Group 3, which was significantly lower than 6.65° in Group 1 (*p* = 0.017) and 6.49° in Group 2 (*p* = 0.002), while no significant difference was found between Groups 1 and 2 (*p* > 0.05). Leg length discrepancy > 2 cm was observed in two patients in Group 1, three patients in Group 2, and one patient in Group 3. The overall mean limb length discrepancy was 5.29 mm (Group 1: 5.89 mm; Group 2: 7.94 mm; Group 3: 3.58 mm), with no significant intergroup difference (*p* > 0.05) (Table 1).

Among the 19 patients who required vascular repair, vascular continuity was restored using different techniques depending on the extent and location of the injury. Primary repair was performed in three cases, end-to-end anastomosis in two cases, and interposition grafting—most commonly with the great saphenous vein—in 13 cases. One patient underwent bypass grafting. Interposition grafting was therefore the most frequently used method, reflecting the severity and complexity of vascular damage in this cohort.

**Fig. 1:**
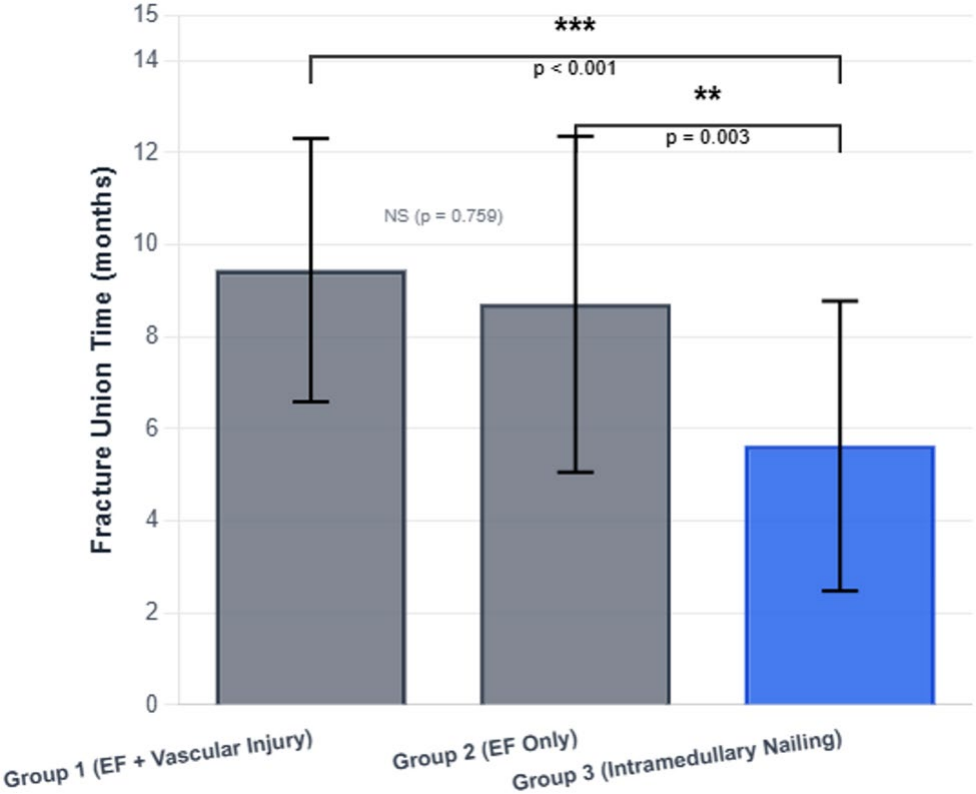
Mean fracture union time by treatment group. Group 1: external fixation with vascular injury (*n* = 19); Group 2: external fixation without vascular injury (*n* = 19); Group 3: intramedullary nailing without vascular injury (*n* = 36). Error bars: ± SD. Group 3 showed significantly faster union than Groups 1 and 2 (***p < 0.001, **p < 0.01; one-way ANOVA with Tukey HSD). EF, external fixation; IMN, intramedullary nailing; VI, vascular injury

The mean fracture union time for the entire cohort was 7.4 ± 3.7 months (31.7 ± 15.6 weeks). Mean union times were 9.5 ± 2.9 months in Group 1, 8.7 ± 3.6 months in Group 2, and 5.6 ± 3.2 months in Group 3 (Tables 1 and 2; Fig. 1). Table 3 presents the odds ratios for delayed union (> 6 months) according to fixation method and vascular injury status.Fracture union occurred significantly earlier in Group 3 than in Groups 1 and 2 (*p* < 0.001), with a mean difference of 1.92–5.25 months between Groups 1 and 3 and 1.28–4.67 months between Groups 2 and 3 (95% confidence intervals). No significant difference in union time was observed between Groups 1 and 2 (*p* = 0.759; 95% CI − 1.71 to 3.05). Figure 1 illustrates these differences in union time, showing clearly shorter healing durations in the intramedullary nailing group compared with both external fixation groups. When delayed union was defined as a union time greater than 6 months, delayed union occurred in 34 of 38 patients (89.5%) treated with external fixation and in 11 of 36 patients (30.6%) treated with intramedullary nailing. External fixation was associated with a significantly increased odds of delayed union compared with intramedullary nailing (OR 19.3, 95% CI 5.5–67.8; *p* < 0.001). Similarly, delayed union was observed in 18 of 19 patients (94.7%) with vascular injury and in 27 of 55 patients (49.1%) without vascular injury, and the presence of vascular injury was associated with higher odds of delayed union (OR 18.7, 95% CI 2.3–149.7; *p* = 0.0003).

**Table 2:** *p*-values for pairwise group comparisons

Comparison	Union time (weeks)	Operation time	Varus–valgus angle	Antecurvatum–recurvatum	Limb length discrepancy	Knee ROM
Group 1 vs. Group 2	0.759	< 0.001	0.993	0.99	0.965	0.638
Group 2 vs. Group 3	0.003	0.014	0.093	0.002	0.116	0.076
Group 1 vs. Group 3	< 0.001	< 0.001	0.252	0.017	0.051	0.063

Group comparisons for continuous variables were performed using one-way analysis of variance (ANOVA), followed by Tukey HSD post-hoc tests where appropriate

**Table 3:** Odds ratios for delayed union (> 6 months) according to fixation method and vascular injury status

	Group A	Group B	Delayed union n/N (%)	OR (95% CI)	*p* value
Fixation method	External fixation (groups 1 + 2)	Intramedullary nailing (group 3)	34/38 (89.5%) vs. 11/36 (30.6%)	**19.3 (5.5–67.8)**	< 0.001
Vascular injury status	Vascular injury present (group 1)	No vascular injury (groups 2 + 3)	18/19 (94.7%) vs. 27/55 (49.1%)	**18.7 (2.3–149.7)**	0.0003

Abreviations: Delayed union was defined as a fracture union time > 6 months. OR, odds ratio; CI, confidence interval

## Discussion

The most important finding of this study was that the method of fixation had a more pronounced impact on fracture healing than the presence of vascular injury, with IMN demonstrating significantly shorter union times and better functional outcomes compared with EF, regardless of vascular status. In the contemporary era, the increasing prevalence of firearm-related injuries continues to underscore the clinical importance of optimizing treatment strategies for gunshot-induced femoral fractures. Within orthopaedic traumatology practice, ongoing debate persists regarding the effects of vascular injury, extensive soft tissue disruption, fasciotomy requirements, surgical exposure during vascular repair, and contamination from retained foreign bodies on fracture healing. IMN and EF remain the two most frequently used fixation methods, and in patients initially managed with EF, the necessity and timing of conversion to IMN have been topics of continued clinical discussion. This study aimed to evaluate the interplay between vascular injury and fixation method on fracture union, and the findings were largely consistent with the available literature.

The comparable demographic characteristics across the groups—including age, sex, BMI, and smoking status—strengthened the internal validity of the analysis by reducing potential confounding effects. This homogeneity enabled a clearer assessment of the independent contributions of vascular injury and fixation technique to fracture healing. Notably, the incidence of peripheral nerve injury in our cohort (18.9%) was higher than the 1%–9% range typically reported for GSW-related femoral trauma [6–9]. This discrepancy may represent differences in injury mechanism, with our cohort possibly including more complex or proximally located wounds. Alternatively, the variation may reflect underdiagnosis in prior studies. Further research is warranted to elucidate the relationship between vascular injury and associated neurological compromise.

In Group 1, the longer operative duration and shorter time-to-surgery reflected the need for urgent vascular repair performed in parallel with fracture stabilization. EF was preferred in patients with vascular injury due to reduced blood loss and shorter initial surgical time; however, conversion from EF to IMN may pose risks to vascular repairs and was therefore not routinely pursued. The Limb Reconstruction System (LRS) provided stable fixation, reducing the necessity for secondary IMN. To maintain group homogeneity, patients who underwent conversion were excluded. Among the excluded patients, conversions occurred early—within a mean of 2.3 days postinjury. Although some studies report benefits of early IMN within 6 h [10], Wiss et al. demonstrated safe outcomes even with delayed IMN at 10–14 days [6]. Although such patients were excluded from the primary analysis in the present study, literature findings support both early and delayed IMN under appropriate conditions. Further quantitative analysis supported these findings. When delayed union was defined as a healing duration greater than 6 months, patients treated with external fixation demonstrated a significantly higher risk of delayed union compared with those treated with intramedullary nailing (OR 19.3, 95% CI 5.5–67.8; *p* < 0.001). Similarly, the presence of vascular injury markedly increased the likelihood of delayed union (OR 18.7, 95% CI 2.3–149.7; *p* = 0.0003). These results reinforce the conclusion that fixation method plays a dominant role in determining healing outcomes, whereas vascular injury appears to exert an additive but less decisive effect when external fixation is used. The magnitude of these odds ratios underscores the clinical relevance of selecting the most biomechanically favorable fixation method whenever patient and wound conditions permit.

IMN is well established as an effective method for low-velocity GSW-related femoral fractures [9]. In the present cohort, EF was used in all vascular injury cases, whereas IMN or EF was selected for non-vascular injuries based on wound contamination and patient condition. Previous studies have shown shorter union times for IMN compared with EF, though some lacked statistical significance [11]. Dabazies reported a union time of 4.8 months using Wagner EF with a mean fixation duration of 8 months [12], while Wiss et al. found union at 23 weeks after IMN [6]. Similarly, Nicholas and McCoy reported healing at 5.5 months with immediate IMN [13]. IMN-treated patients in the present study exhibited union times comparable to those reported previously, albeit slightly longer. IMN was associated with significantly faster union compared with EF (*p* < 0.001), likely attributable to intramedullary reaming promoting autologous grafting. This difference was supported by large effect sizes (Cohen’s d = 1.25, 95% CI [0.71–1.78] for Group 1 vs. 3; d = 0.93, 95% CI [0.41–1.44] for Group 2 vs. 3). The lack of a significant difference between vascular and non-vascular EF groups (Cohen’s d = 0.23) suggests that vascular injury alone may not substantially delay healing when EF is employed.

Patients treated with LRS EF in this study demonstrated longer healing durations than those historically reported, likely attributable to the practice of maintaining fixation until complete clinical and radiographic union. Although EF-treated patients with vascular injury (Group 1) showed longer healing times than those without vascular injury (Group 2), the difference did not reach statistical significance. The overall mean union time of 7.4 ± 3.6 months was consistent with findings by Abdioglu et al., who reported a mean healing time of 7.4 months across various fixation methods [14].

Functional outcomes also favored IMN, as knee ROM was significantly greater in Group 3 relative to both EF groups (*p* = 0.001). Literature supports these findings, reporting knee ROM values between 125° and 132° following IMN [9, 10, 13], while EF methods—including LRS and Ilizarov—yielded more modest ROM values [11–15]. Although Ilizarov fixation may initially result in stiffness, fasciotomy at pin sites and dedicated rehabilitation can improve outcomes.

Limb-length discrepancy findings in this study (mean 5.29 mm) were consistent with previously reported values of 4–6 mm in IMN and EF-treated patients [11, 15, 16]. Additionally, varus–valgus deformities were similar among groups, while IMN demonstrated significantly lower antecurvatum–recurvatum deformities (*p* = 0.002), likely reflecting the greater mechanical stability offered by IMN compared with EF.

This study has several limitations. Its retrospective design limits the ability to control for all confounders, although multivariate regression analysis identified the fixation method as the only independent predictor of union time (*p* < 0.001), whereas age, BMI, smoking, and injury side did not significantly affect healing. The exclusion of 46 patients—necessary for group homogeneity—may have introduced selection bias. Differences in fracture distribution across groups and unequal group sizes may have influenced comparative outcomes. Future research should include prospective, multicenter studies with larger and more evenly distributed cohorts, detailed vascular injury classification, standardized timing of surgical intervention, and monitoring of additional confounding variables such as infection and injury severity.

## Conclusion

Intramedullary nailing resulted in markedly shorter union times, better alignment, and superior knee motion compared with external fixation in gunshot-related femoral fractures. Although vascular injury contributed to prolonged healing among externally fixated patients, this effect was less decisive than the fixation method. Delayed union risk was significantly higher with external fixation and in the presence of vascular injury, consistent with their respective odds ratios. Fixation method remained the only independent predictor of union time. Prospective, larger-scale studies are needed to validate these findings and refine treatment algorithms.

## Data Availability

The datasets generated and/or analyzed during the current study are available from the corresponding author on reasonable request.
